# Supplementary material for: Addressing Critical Issues Related to Storage and Stability of the Vault Nanoparticle Expressed and Purified from Komagataella phaffi
Source: Int J Mol Sci. 2023 Feb 20;24(4):4214. doi: 10.3390/ijms24044214 (PMC9959619; doi:10.3390/ijms24044214)
Supplement: Supplementary file 1 [file ijms-24-04214-s001.zip › ijms-2147797-supplementary.pdf]

Supplementary information

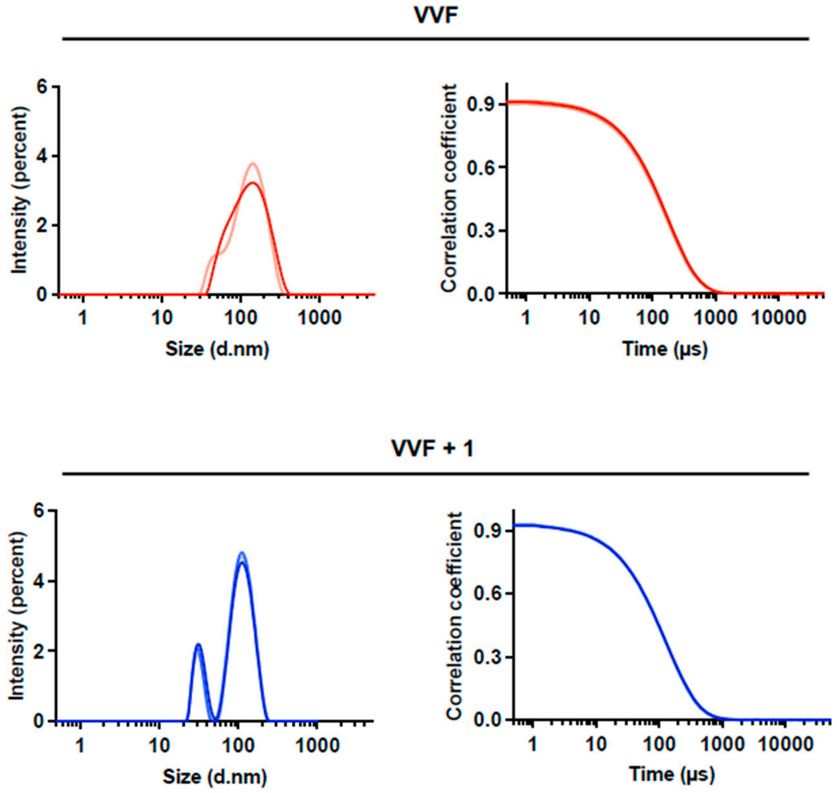

Figure S1. DLS of the 20,000 x g supernatants of freshly purified vault from the SEC void-volume fraction (VVF) and the following one (VVF+1). Intensity-weighted particle size distribution and autocorrelation function are presented.

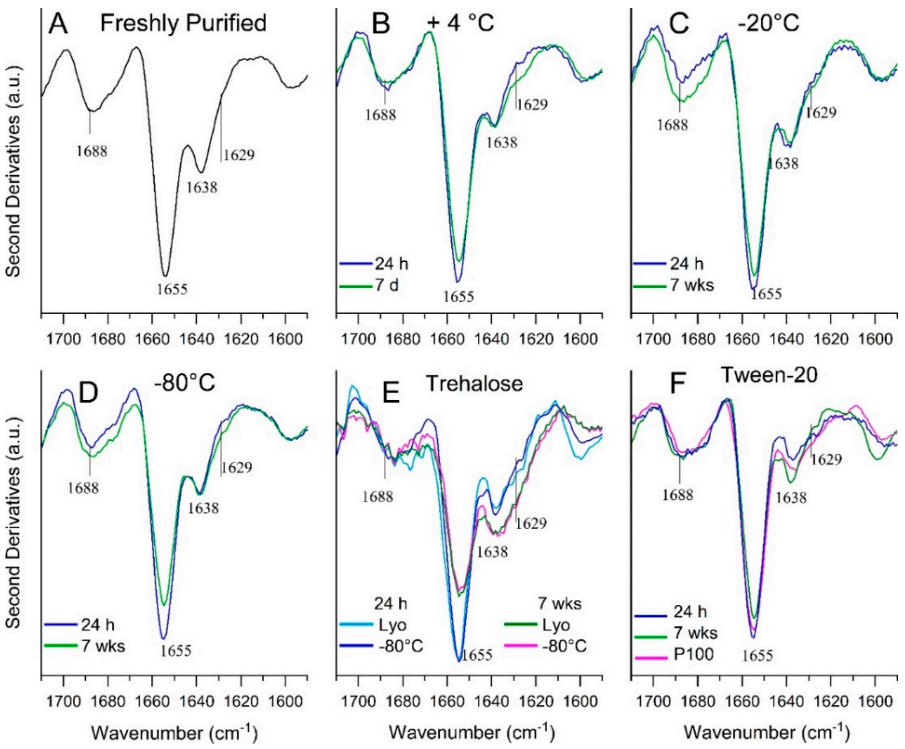

Figure S2. FTIR analysis of whole samples of vault protein preparations. Analyses were performed prior to protein centrifugation and otherwise as described in the legend to Figure 4, except that after a 7-weeks incubation in 0.05% Tween-20 under frozen conditions, the sample was thawed, centrifuged at 100,000 x g for 2 h, and the pellet analyzed (P100, panel F).

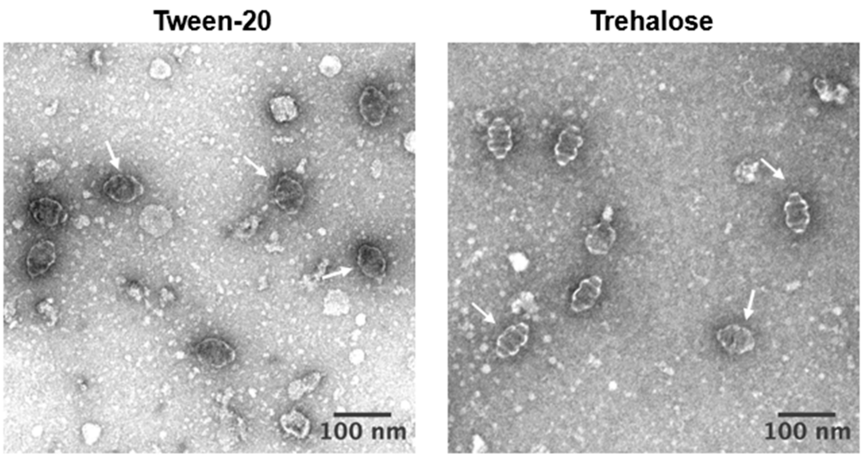

Figure S3. TEM images of vault nanoparticles stained by uranyl acetate, as detected in samples frozen at -80 °C in the presence of either 0.05% Tween 20 or 10 mg/ml trehalose and stored for 7 weeks. The fractions were analyzed after centrifuging at 20,000 x g for 20 min and discarding the pellet. Some vault particles are indicated by white arrows.

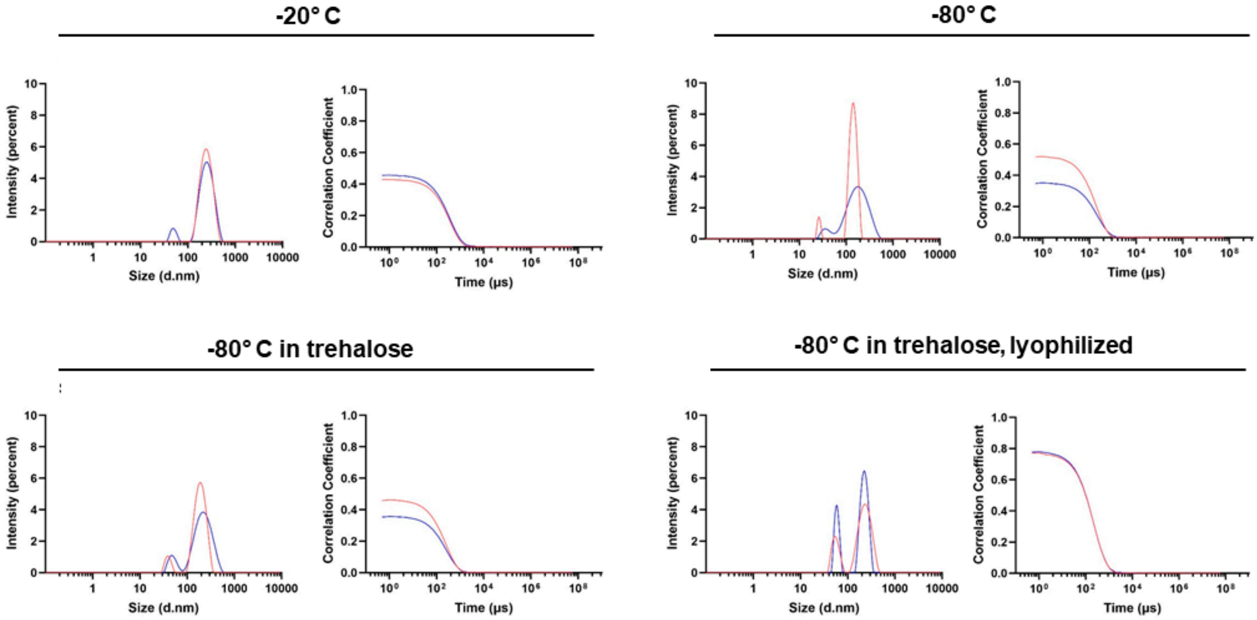

Figure S4. DLS of the 20,000 x g supernatants of vault samples incubated for 7 weeks and otherwise as indicated in the respective panels. Intensity-weighted particle size distribution and autocorrelation function are presented. In each panel, two replicates of the same measurement are shown.
